# Contracted Pelvis

**Published:** 1873-11

**Authors:** 


CONTRACTED PELVIS.—Dr. Angus McDonald, of Edinburg, concludes: 1st—It seems exceedingly doubtful if the operation of inducing premature labor ought ever to be employed in cases of contracted pelvis. 2d—That turning does not present any proved advantage over long forceps in cases of contracted flat pelvis, (as far, at least, as my experience and my reading enable me to form an opinion), and is undoubtedly more dangerous to the child. That it is entirely unsuitable when the contraction is general, being much more dangerous to the mother than long forceps, or any of the higher operations. 3d—That in contracted pelvis, as a general rule, it is on the whole safer to let the case go on to the full term of utero-gestation, and then give the patient a fair trial, so as to ascertain what nature is likely to accomplish unaided, without waiting so long as to allow the mother to run unnecessary risk. Then, in case there is room for the introduction of forceps, they ought to be applied, and delivery attempted by their means. If this is impossible, then delivery ought to be effected by cephalotripsy, craniotomy, or cesarean section.

Dr. Matthews Duncan agreed in most points with the views expounded by Dr. McDonald.

Dr. J. Matthews Duncan, while producing artificial rotation of the head in a case of narrow pelvic outlet, made with his finger an impression in the parietal bone to the depth of about half the thickness of his finger. The result was slight, short, but very frequently repeated, epileptiform seizures, which lasted for some time after the digital impression had disappeared. Dr. Steele, of Liverpool, suggested that rotation of the fetal head at the outlet might be effected by the forceps more readily, and with less risk of injury, than by continued pressure on one point of the cranium by the finger.

